# Innate Lymphoid Cells Activation and Transcriptomic Changes in Response to Human Dengue Infection

**DOI:** 10.3389/fimmu.2021.599805

**Published:** 2021-05-17

**Authors:** Tiraput Poonpanichakul, Wilawan Chan-In, Anunya Opasawatchai, Fabien Loison, Oranart Matangkasombut, Varodom Charoensawan, Ponpan Matangkasombut, DENFREE Thailand, Anavaj Sakuntabhai

**Affiliations:** ^1^ Department of Microbiology, Faculty of Science, Mahidol University, Bangkok, Thailand; ^2^ Systems Biology of Diseases Research Unit, Faculty of Science, Mahidol University, Bangkok, Thailand; ^3^ Chakri Naruebodindra Medical Institute, Faculty of Medicine Ramathibodi Hospital, Mahidol University, Samut Prakan, Thailand; ^4^ Department of Clinical Pathology, Faculty of Medicine Vajira Hospital, Navamindradhiraj University, Bangkok, Thailand; ^5^ Faculty of Dentistry, Mahidol University, Bangkok, Thailand; ^6^ Department of Microbiology and Research Unit on Oral Microbiology and Immunology, Faculty of Dentistry, Chulalongkorn University, Bangkok, Thailand; ^7^ Laboratory of Biotechnology, Chulabhorn Research Institute, Bangkok, Thailand; ^8^ Department of Biochemistry, Faculty of Science, Mahidol University, Bangkok, Thailand; ^9^ Integrative Computational BioScience Center (ICBS), Mahidol University, Nakhon Pathom, Thailand

**Keywords:** Dengue, viral infection, innate lymphoid cells, ILCs, immune response to dengue, innate immunity, RNA-seq, transcriptome

## Abstract

**Background:**

Dengue virus (DENV) infection has a global impact on public health. The clinical outcomes (of DENV) can vary from a flu-like illness called dengue fever (DF), to a more severe form, known as dengue hemorrhagic fever (DHF). The underlying innate immune mechanisms leading to protective or detrimental outcomes have not been fully elucidated. Helper innate lymphoid cells (hILCs), an innate lymphocyte recently discovered, functionally resemble T-helper cells and are important in inflammation and homeostasis. However, the role of hILCs in DENV infection had been unexplored.

**Methods:**

We performed flow cytometry to investigate the frequency and phenotype of hILCs in peripheral blood mononuclear cells from DENV-infected patients of different disease severities (DF and DHF), and at different phases (febrile and convalescence) of infection. Intracellular cytokine staining of hILCs from DF and DHF were also evaluated by flow cytometry after *ex vivo* stimulation. Further, the hILCs were sorted and subjected to transcriptome analysis using RNA sequencing. Differential gene expression analysis was performed to compare the febrile and convalescent phase samples in DF and DHF. Selected differentially expressed genes were then validated by quantitative PCR.

**Results:**

Phenotypic analysis showed marked activation of all three hILC subsets during the febrile phase as shown by higher CD69 expression when compared to paired convalescent samples, although the frequency of hILCs remained unchanged. Upon *ex vivo* stimulation, hILCs from febrile phase DHF produced significantly higher IFN-γ and IL-4 when compared to those of DF. Transcriptomic analysis showed unique hILCs gene expression in DF and DHF, suggesting that divergent functions of hILCs may be associated with different disease severities. Differential gene expression analysis indicated that hILCs function both in cytokine secretion and cytotoxicity during the febrile phase of DENV infection.

**Conclusions:**

Helper ILCs are activated in the febrile phase of DENV infection and display unique transcriptomic changes as well as cytokine production that correlate with severity. Targeting hILCs during early innate response to DENV might help shape subsequent immune responses and potentially lessen the disease severity in the future.

## Introduction

Dengue virus (DENV) infection is a serious public health threat, especially in tropical and subtropical areas. This important mosquito-borne virus infects approximately 390 million people annually (1). Clinical manifestations range from asymptomatic, mild dengue fever (DF) to severe life-threatening dengue hemorrhagic fever (DHF) and dengue shock syndrome (DSS) (2). Currently, there is no specific treatment. The only licensed vaccine, Dengvaxia, showed limited efficacy and inadvertently increased hospitalization rate in children and dengue-naive vaccinees (3–5). The high prevalence, absence of specific treatment and lack of effective vaccine result in prominent global burden, clinically, and economically. This is in part due to the inadequate understanding of immune responses to DENV infection.

Complex interactions between DENV and host immune responses lead to the various outcomes of the infection. While protective immune response is required for viral clearance and resolution of the infection, detrimental response results in increased viral propagation, cytokine storm, plasma leakage, and severe disease outcome (6–9). DENV is a single stranded positive-sense RNA virus in the *Flaviviridae* family. In humans, there are 4 DENV serotypes. Secondary heterotypic infection is associated with an increased chance of developing severe disease (10, 11), likely due to pathogenic memory T and B cell response from previous infection known as T cell antigenic sin (9, 12, 13) and antibody-dependent enhancement (14, 15). Innate and innate-like responses to DENV infection are not only crucial as the first line of defense but also influence subsequent adaptive T and B cell responses (16). Beside the role of innate-like T cells, NKT (17–19) and MAITs (20, 21), several lines of evidence suggested the important roles of various innate responses in DENV infection and viral evasion strategies, in particular type I IFN (22–24), monocytes, macrophages and dendritic cells (25), mast cells (18, 26), as well as NK cells (27). However, the roles of innate lymphoid cells (ILCs), a very important and most recently discovered innate immune cells, in DENV infection has never been investigated.

ILCs are innate counterparts of T cells that can respond rapidly, orchestrate early innate responses, and shape subsequent adaptive responses (28–35). Unlike T cells, they do not express T-cell receptors, thus do not respond in an antigen-specific manner. ILCs comprise NK cells, helper ILCs (hILCs), and lymphoid tissue inducers (LTi). Helper ILCs are classified into ILC1, ILC2, and ILC3, based on expression of major transcription factors and their signature cytokines, which resemble those of Th1, Th2 and Th17/Th22 cell subsets. In general, ILC1 produces IFN-γ; ILC2 produces IL-4, IL-5 and IL-13; and ILC3 produces IL-17A and/or IL-22 (32, 33, 36, 37). Recent evidence also showed the plasticity of ILCs that enable them to promptly respond to environmental changes (38). hILCs have been shown to play both protective and detrimental roles in various diseases, including allergy (39–41), autoimmunity (42–44), cancers (45–48), inflammation and infectious diseases caused by various pathogens including viruses (49–55).

While the critical roles of hILCs have been demonstrated in several viral infections, most studies were done in murine models. These include diverse roles in host protection, immunopathology, and tissue homeostasis in influenza A virus (IAV) (56–60), respiratory syncytial virus (RSV) (61–63), rhinovirus (64, 65), herpes simplex virus (HSV) (66), rota virus (67), and mouse cytomegalovirus (MCMV) (68–70). Because mouse and human hILCs differ significantly (71), study in the human system is critical. Using human hILCs co-cultured with viruses *in vitro*, hILCs were shown to respond to rhinovirus (72), IAV (56, 58), and human cytomegalovirus (HCMV) (73). However, the study of hILCs in natural viral infection in humans has been limited to those of HIV infection (74).

Here, we investigated the potential roles of hILCs in natural human DENV infection using clinical samples from a well-characterized DENV-infected patient cohort. Flow cytometric analysis showed marked hILCs activation during the febrile phase which diminished at convalescence in both DF and DHF, while hILC number and subset composition remained unchanged. Upon ex vivo stimulation, hILCs from febrile phase DHF produced more cytokine than those of DF. Furthermore, global gene expression analysis revealed upregulation of different sets of genes in the febrile phase of DF and DHF patients. These results suggested that hILCs play a role in response to febrile phase of DENV infection and that diverged hILCs functional responses were associated with different clinical outcomes of the infection. A better understanding of hILCs within the complex host-viral interaction in the pathogenesis of DENV infection may contribute to future development of effective preventative and therapeutics approaches.

## Materials and Methods

### Ethical Statement

This study used human samples collected from Dengue Research Framework for Resisting Epidemics in Europe (DENFREE) (75). The DENFREE (Thailand) (76) study was approved by the Institutional Review Board of Faculty of Medicine Vajira Hospital (No.015/12) and the Faculty of Tropical Medicine Mahidol University (TMEC 13-041). All subjects or their legal guardians signed written informed consent prior to study participation. The use of archived DENFREE samples in this study was approved by the ethical committee of the Faculty of Medicine, Ramathibodi hospital (COA.MURA2019/603).

### Clinical Samples

Blood samples were collected from DENV-infected patients who presented with febrile illness with confirmed presence of DENV RNA in plasma by RT-PCR, as previously described (77, 78). The DENV-infected patients were classified according to WHO 1997 classification criteria into dengue fever (DF) and dengue hemorrhagic fever (DHF).

**Figure 1:**
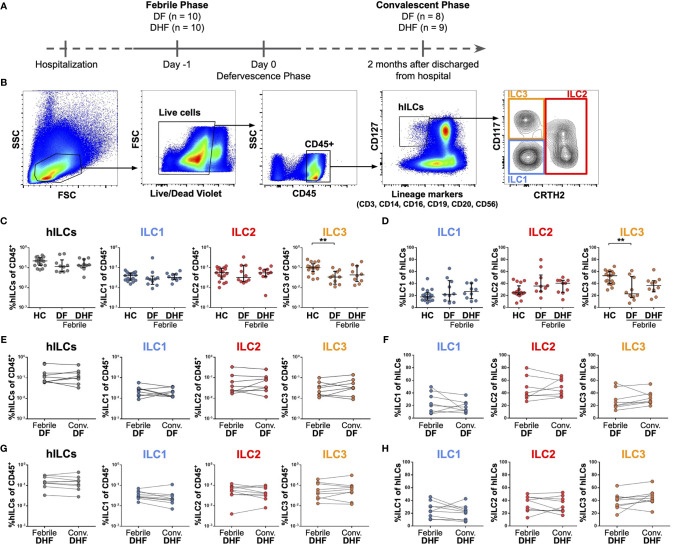
Frequencies of hILCs during the course of DENV infection. **(A)** Study design **(B)** Flow cytometry analysis gating strategy for hILCs and their subsets from PBMC. **(C, D)** Percentage of total hILCs and hILC subsets in febrile phase of dengue fever (DF), dengue hemorrhagic fever (DHF) patients and healthy donors (HC). ILC subsets frequencies were determined by percentage of CD45+ lymphocyte **(C)** or total hILCs **(D)**. **(E, F)** Percentage of total hILCs and ILC subsets of matched samples at febrile and convalescent phases of DF patients. ILC subset frequencies were determined by percentage of CD45+ lymphocyte **(E)** or total hILCs **(F)**. **(G, H)** Percentage of total hILCs and ILC subsets of matched samples at febrile and convalescent phases of DHF patients. ILC subsets frequencies were determined by percentage of CD45+ lymphocyte **(G)** or total hILCs **(H)**. The results were presented as Median ± IQR. Data were analyzed using Mann-Whitney test **(C, D)** or Wilcoxon matched-pairs signed rank test **(E–H)** (**p < 0.01).

**Figure 2:**
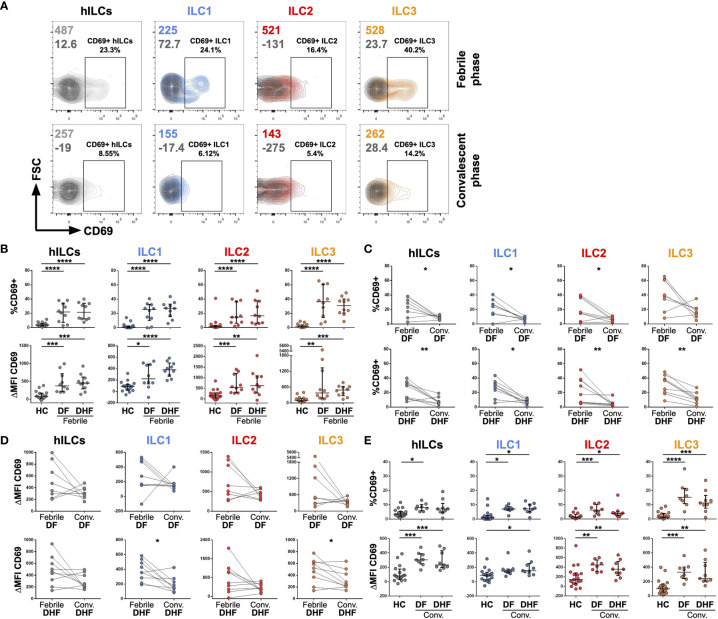
Activation of hILCs during the febrile phase of DENV infection. **(A)** Representative contour plot of CD69 expression (light grey, blue, red, and orange) as compared with isotype control (dark grey) on total hILC and each hILC subsets in febrile phase and convalescent phases. **(B)** Percentage of CD69+ (upper panel) and ΔMFI (CD69 MFI - isotype control MFI) (lower panel) for total hILCs and each hILC subset in febrile phase of dengue fever (DF) patients and dengue hemorrhagic fever (DHF) patients compared to healthy donors (HC). **(C)** Percentage of CD69+ and **(D)** ΔMFI for total and hILC subsets of febrile phase compared to matched convalescent of DF (upper panel) and DHF (lower panel). Each line connected data of the same patient between two timepoints. Wilcoxon matched-pairs signed rank test was used for statistical comparison, *p* < 0.05 was considered as a statistically significant difference. **(E)** Percentage of CD69+ (upper panel) and ΔMFI (lower panel) for total and hILC subsets in convalescent phase of DF and DHF patients compared to healthy donors (HC). The results were presented as Median ± IQR. Data were analyzed using Mann-Whitney test **(B, E)** or Wilcoxon matched-pairs signed rank test **(C, D)** (*p < 0.05, **p < 0.01, ***p < 0.001, ****p < 0.0001).

**Figure 3:**
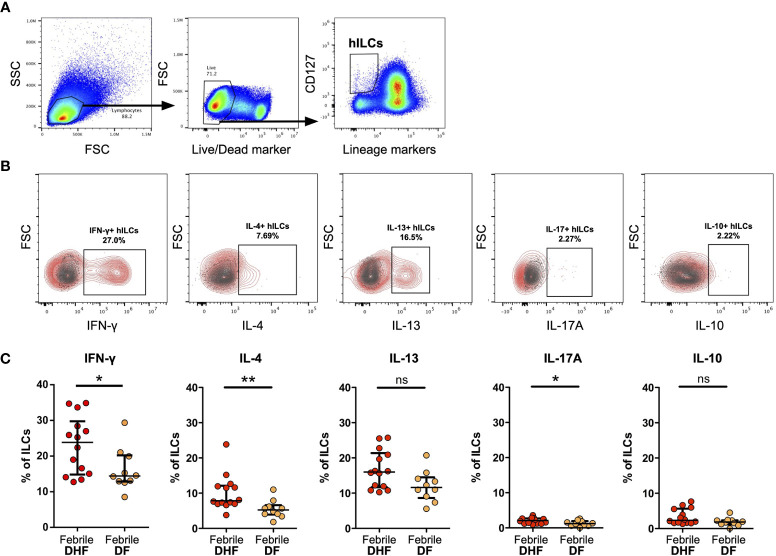
Intracellular cytokine staining of hILCs from febrile DF and DHF after PMA/ionomycin *ex vivo* stimulation **(A)** Flow cytometry analysis gating strategy of total hILCs from PBMC. **(B)** Representative contour plots of IFN-γ, IL-4, IL-13, IL-17A, and IL-10 expression (red) overlaid on FMO control (grey). **(C)** Percentage of IFN-γ, IL-4, IL-13, IL-17A, and IL-10 expression of hILCs in febrile phase of DF and DHF patients. The results were presented as median ± IQR. Data were analyzed using Mann-Whitney test (*p < 0.05, **p < 0.01; ns, not significant).

**Figure 4:**
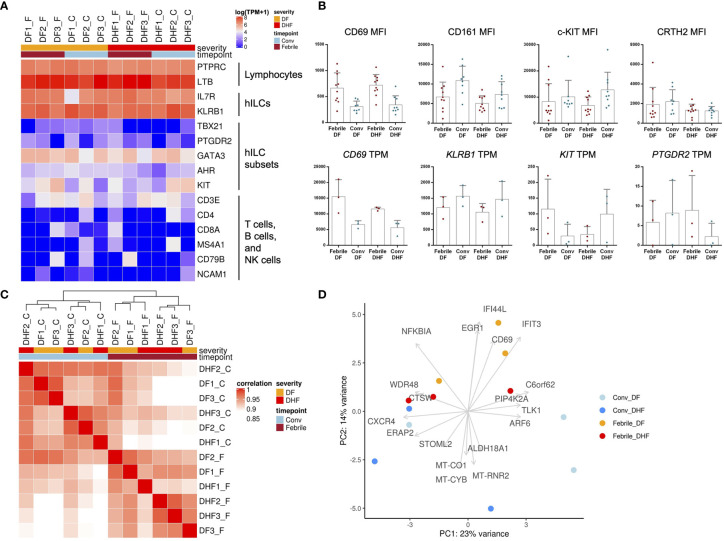
Transcriptome analysis of hILCs from DENV-infected patients. **(A)** Heatmap showing expression level (in TPM) of known hILCs and their subsets’ signature genes as compared to those of T cells, B cells and NK cells in each sample arranged according to disease severity (DF, DHF) and timepoints (febrile, convalescence). **(B)** Bar plots show mean MFI from previous flow cytometry experiment (upper panel) and gene expression levels in TPM (lower panel) for CD69, CD161 (*KLRB1*), c-kit (CD117, *KIT*), and CRTH2 (CD294, *PTGDR2*). **(C)** Correlation of global transcriptome profiles of hILCs in all samples. Unbiased hierarchical clustering shows high correlation amongst samples according to timepoints (febrile phase clustered together and away from convalescence). **(D)** PCA plot with top contribution genes for PC1 and PC2 shows separation between febrile and convalescent phase on PC2 which were contributed by the expression of *CD69*, *EGR1*, *IFIT3*, *NFKBIA*, and *IFI44L*. Sample number indicated on top of heatmap; DF1_F (DF sample number 1, febrile phase), DF1_C (DF, sample number 1, convalescent phase).

**Figure 5:**
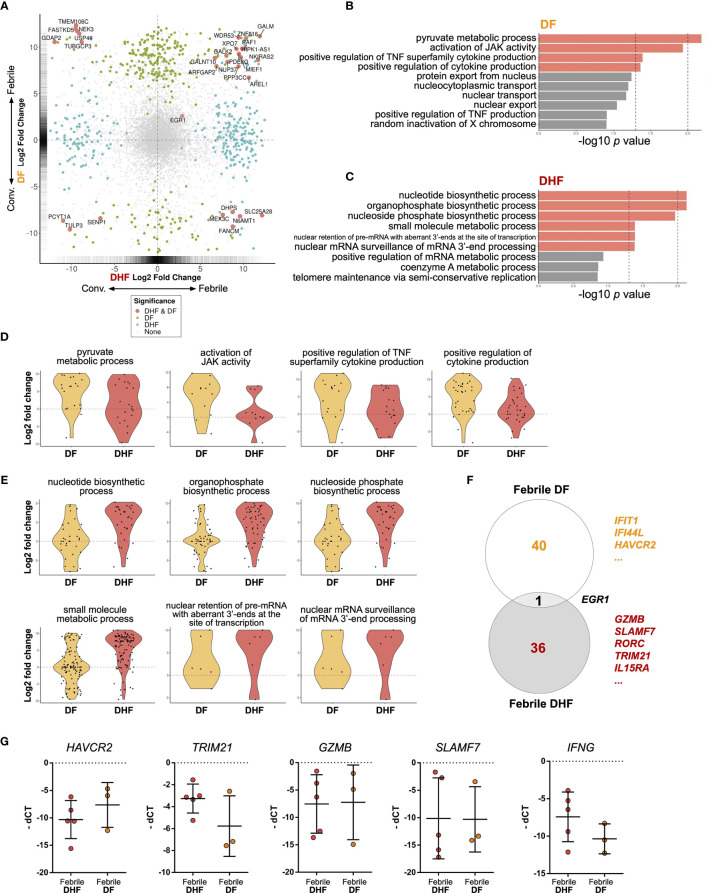
Differential gene expression analysis of hILCs between febrile and convalescent phase of DENV infection **(A)** Plot comparing the fold change of DF (febrile over convalescent phase) (Y axis) to the fold change of DHF (febrile over convalescent phase) (X axis) depicts transcripts differentially expressed between disease severity and timepoint. Colored dots denote transcripts that are differentially expressed at least 2-fold higher as well as adjust p value less than 0.01. Blue or green colored dots denote differentially expressed in DF or DHF respectively. Red colored dots denote transcripts that are differentially expressed by both DF and DHF. **(B, C)** Bar plots show significant GO term enrichment from DE genes comparing febrile and convalescent phases in **(B)** DF and **(C)** DHF patients. **(D, E)** Violin plots show fold change of expression levels of matched samples between febrile and convalescent phases. Each dot represents a gene in the designated GO term by each patient. **(D)** show enriched GO term in DF patients and **(E)** shows GO term enriched in DHF patients. **(F)** Venn diagram shows number of differentially expressed genes in DF (febrile over convalescent phase), DHF (febrile over convalescent phase) and both after shrinkage algorithm ‘apeglm’ was applied. Immune-related genes are listed beside the diagram. **(G)** Expression levels of *HAVCR2*, *TRIM21*, *GZMB*, *SLAMF7*, and *IFNG* by quantitative PCR (delta Ct value (dCt) = Ct value of *ACTB* - Ct value of interested gene). Bar showing median ± IQR.

Samples from two timepoints were evaluated in this study (**Supplementary Table 1**). Febrile phase samples were collected one day before fever subsided to represent the febrile phase of DENV infection. Convalescent phase samples were collected two months after hospital discharge (**Figure 1A**). Peripheral blood mononuclear cells (PBMCs) were isolated using density gradient centrifugation (Lymphoprep, STEMCELL Technologies, 07851). Aliquots of PBMCs were then cryopreserved in freezing media (90% FBS, 10% DMSO) in liquid nitrogen until used. PBMCs from 10 DF and 10 DHF patients at both febrile and matched convalescent phases (when available) were used for surface flow cytometric experiments (**Figures 1**, **2**). A subset of these samples (3 DF and 5 DHF patients) was used for hILC cell sorting experiment in which 3 DF and 3 DHF samples proceeded to RNA sequencing (RNA-seq) experiment (**Figures 4**, **5**). The cDNA from sorted hILCs (3 DF and 5 DHF patients) were used for qPCR experiment. A different set of PBMCs at febrile phase (10 DF and 14 DHF patients) were used for intracellular cytokine staining experiment (**Figure 3**).

### Helper ILCs Phenotypic Analysis by Flow Cytometry

Cryopreserved PBMCs were thawed at 37°C with warm RPMI-1640 (Gibco, 11875119) supplemented with 10% FBS (Gibco, 10099141). After washing twice with RPMI, cell viability was assessed using trypan blue exclusion assay. Cell viability of all samples in this study exceeded 90%. PBMCs were stained with LIVE/DEAD fixable violet (Invitrogen, L34963) according to the manufacturer’s protocol. Subsequently, Fc blocking with Human TruStain FcX™ (Biolegend, 422302) and cell surface staining were performed. The fluorophore-conjugated antibodies for cell surface staining comprise of lineage cocktail (CD3, CD14, CD16, CD19, CD20, CD56) (FITC), CD117 (PE), CRTH2 (Alexa Fluor 647), CD127 (APC/Cy7), CD45 (V500), and CD69 (Alexa Fluor 700) (for list of antibodies see **Supplementary Table 2**). Fluorescence minus one (FMO) and isotype controls (mIgG1-Alexa Fluor 700, BD Biosciences) were used to evaluate CD69 expression. Surface staining was performed on ice for 30 minutes in FACS Buffer (2% FBS and 0.25 mM EDTA in PBS). Stained samples were then acquired with the CytoFLEX flow cytometer (Beckman Coulter) using CytExpert version 2.3. FlowJo version 10 (FlowJo, LLC) and GraphPad Prism version 7 were used for data analysis. hILCs were identified within the lymphocyte region on the basis of their forward and side scatter profiles (FSC^low^ and SSC^low^) in conjunction with CD45^high^SSC^low^ after excluding doublets and dead cells. Total hILCs were gated as CD45^+^Lin^−^CD127^+^ cells. The hILC subsets were then identified within total hILCs as follows: CD117^-^hILCs as ILC1, CD161^+^CRTH2^+^hILCs as ILC2, and CD117^+^hILCs as ILC3 (**Figure 1B**).

### Intracellular Cytokine Staining of hILCs

Cryopreserved PBMCs were thawed and processed as described above. Fc blocking with Human TruStain FcX™ (Biolegend, 422302) was performed according to the manufacturer’s protocol. Cell surface staining with lineage cocktail (FITC) and CD127 (APC/Fire750) was performed at RT for 15 minutes. PBMCs were then stimulated with PMA (50 ng/ml) and Ionomycin (1 ug/ml) for 4 hours with protein transport inhibitor, BD GolgiPlug (BD, 555029). PBMCs were stained with Zombie Violet Fixable Viability Kit (Biolegend, 423114) before undergoing intracellular cytokine staining with BD Cytofix/Cytoperm™ (BD, 554714) according to manufacturer’s protocol. IFN-γ (PE), IL-4 (BV510), IL-13 (PerCP/Cy5.5), IL-17A (Alexa Fluor 700), and IL-10 (Alexa Fluor 647) were used for intracellular cytokine staining for 30 minutes on ice (see **Supplementary Table 2** for a list of antibodies used). Stained samples were then acquired and analyzed with CytExpert version 2.3 and GraphPad Prism version 7, in comparison to fluorescence minus one (FMO) control.

### Helper ILC Sorting

PBMCs from 3 DF and 5 DHF patients at febrile and convalescent phases were sorted for hILCs using a FACS Aria III instrument (BD Biosciences) for hILCs RNA-seq and qPCR experiments. PBMCs were stained with Lineage cocktail antibodies (CD3, CD14, CD16, CD19, CD20, CD56) (FITC) and CD127 (APC/Cy7). After staining, cells were washed and resuspended in a cold FACS buffer (2% FBS and 0.25 mM EDTA in PBS). Immediately before sorting, cells were filtered through a 35-μm strainer. Helper ILCs were gated as Lin^-^CD127^+^ cells within the lymphocyte gate (FSC^low^SSC^low^) after exclusion of doublets and sorted directly into in 96-well plates with 4 μl of lysis buffer composed of 0.4% Triton-X 100 (Calbiochem, 648466), recombinant RNase inhibitor (Clonetech, 2313B), and 10 mM dNTP mix (Bioline, 39053). Sorting mode was set to single cell resolution in order to gain the highest purity. Fifty hILCs were sorted into each well. Approximately 3-5 wells were obtained per sample. Plates were immediately spun down after sorting, then sealed and snapped frozen with dry ice before moving to -80°C freezer for storage until further processing within 2-3 days.

### Microscaled RNA-Seq

The generation of full-length transcriptomes from a low number of cells per sample was performed based on the SMART-seq2 protocol (79), with modifications. Briefly, mRNA was captured using poly-dT oligonucleotides and reverse-transcribed into full-length cDNA using the described template-switching oligonucleotide and SMARTScribe (Clonetech, 639537). cDNA was amplified by PCR (PCRmax Alpha Cycler 2) for 14 cycles using the KAPA HiFi HotStart Readymix (Roche, KK2601, 07958927001) and then purified using AMPure XP (Beckman Coulter, A63881) magnetic beads at 0.8:1 (vol/vol) ratio. For each well, cDNA quality was assessed with Agilent Tapestation (expected peak ~ 1.5-2 kb). cDNA profiles containing short fragments (< 500 bp), possibly due to RNA degradation, were excluded. cDNA from three wells of the same sample were pooled and normalized to 300 pg/ul for subsequent library construction using the Nextera XT library preparation kit (Illumina, FC-131-1096) and the index kits (Illumina, FC-131-2001). Each library’s size was assessed by Agilent Tapestation. The libraries were sequenced using the Illumina HiSeq platform (Macrogen, South Korea) with paired-end 150-bp read length and coverage of approximately 30 million reads per sample.

### Quantitative PCR Analysis of Sorted hILCs

RNA from sorted hILCs were reverse transcribed and preprocessed into cDNA as described above. cDNA was normalized to 5 ng/ul for each PCR reaction. Quantitative PCR reactions were prepared with Q5 High-Fidelity 2X Master Mix (NEB, M0492L) and SYBR green I (Roche, 11988131001). Oligos used for qPCR are listed in **Supplementary Table 3**. All qPCR reactions were performed using the Rotor-Gene Q real-time cycler (Qiagen). The specificity of the reaction was verified by melting curve analysis. Delta Ct value for each gene is compared to *ACTB*.

### Bioinformatics Analyses

Nextera adapter sequences were removed using Trimmomatic version 0.36 (80). Trimmed reads were mapped and aligned using HISAT2 (81), with GRCh38 as the reference genome. Normalized relative transcript abundances as Transcripts Per Kilobase Million (TPM) were obtained using StringTie (82). Raw read counts were obtained with the HTseq-count version 0.6.1p1 (83).

After removal of absent features (zero counts in all samples), the raw counts were then imported to DESeq2 version 1.24.0 (84) to identify differentially expressed (DE) genes. DE analysis was performed by comparing the febrile phase and convalescent phase samples of the same individuals (“paired samples” in the design formula of DESeq2), so the biases between the donors were internally normalized. Wald-test *p* values were adjusted for multiple testing using the Benjamini-Hochberg method, and genes with adjusted *p* values less than 0.01 and with log2 fold changes greater than 2 or less than -2, were considered significantly differentially expressed between the two phases. Because of the low sample numbers, which may cause high variability within each sample group, shrinkage estimator ‘apeglm’ was applied to re-estimate the log2 fold change. Apeglm estimates the effect size more accurately, especially when read counts are low and highly variable (85). Genes with adjusted *p* values less than 0.01 and with re-estimated log2 fold changes greater than 2 or less than -2, were considered statistically significant. Functional gene set analysis was assessed using the Gene Ontology (GO) biological process analysis with gprofiler (86). ComplexHeatmap (87) was used to generate heatmap plots for visualization.

## Results

### Frequency of hILCs Did Not Change During Febrile Phase of DENV Infection

To investigate the role of hILCs during DENV infection, we first examined the frequency of hILCs and hILC subsets in PBMC from DF and DHF patients during febrile phase of DENV infection, as compared to those at the convalescence and also to healthy controls (HC), using flow cytometry (**Figures 1A, B**). There was no significant difference in terms of total hILC frequency among febrile DF (median 0.12, IQR 0.07 - 0.16), DHF (median 0.14, IQR 0.12 - 0.23), and HC (median 0.22, IQR 0.13 - 0.29) (**Figure 1C**). No obvious change in hILC subset distribution was observed, with the exception of a lower percentage of ILC3 in the febrile DF patients (% of CD45+ median 0.03, IQR 0.02 - 0.06; % of hILCs median 22.80, IQR 18.33 - 45.23), when compared to HC (% of CD45+ median 0.10, IQR 0.08 - 0.12; % of hILCs median 53.10, IQR 40.30 - 60.10) (*p* < 0.01) (**Figures 1C, D**). Furthermore, the frequency of total hILCs and hILC subsets were not significantly different when compared between the febrile phase of DENV infection and the convalescence of the same patient, regardless of disease severity (**Figures 1E–H**). Thus, hILC frequency did not change during DENV infection.

### Helper ILCs Were Activated During Febrile Phase of DENV Infection

To investigate whether hILCs were activated during DENV infection, the expression of CD69 (in comparison to FMO control) on hILCs was investigated (**Figure 2A**). The percentage of CD69+ hILCs, when compared to that of the healthy donors (median 3.56, IQR 2.38 - 5.04), were significantly higher in both febrile DF (median 21.95, IQR 10.72 - 32.13, *p* < 0.001) and febrile DHF (median 21.55, IQR 12.58 - 33.05, *p* < 0.001) (**Figure 2B**, upper panel). The changes in CD69 mean fluorescence intensity (ΔMFI) showed a similar result (**Figure 2B**, lower panel). However, the expression level of CD69 was not different between hILCs of DF and DHF patients at the same time points. Expression of CD69 on ILC1 (DF median 25.50, IQR 13.73 - 31.03; DHF median 27.25, IQR 18.85 - 31.93; HC median 0.95, IQR 0.00 - 2.17), ILC2 (DF median 14.70, IQR 7.36 - 31.40; DHF median 16.65, IQR 6.67 - 35.13; HC median 1.44, IQR 0.80 - 2.40), and ILC3 (DF median 36.35, IQR 19.40 - 55.65; DHF median 30.90, IQR 21.75 - 38.45; HC median 1.30, IQR 0.86 - 3.58) were significantly higher during febrile DF and DHF when compared to HC.

In addition, analysis of hILC activation kinetics by comparing expression levels of CD69 between the febrile and convalescent samples from the same patient showed a decrease in hILC activation when disease subsided (**Figures 2C, D**, left most column). Further analysis on all hILC subsets show similar results of decreased activation in the convalescence (**Figures 2C, D**). Interestingly, low level of hILC activation seemed to persist in the convalescent phase as their CD69 expression was still higher than those of healthy donors (**Figure 2E**). These results suggest that hILCs were highly activated during the febrile phase of DENV infection, and the activation diminished to a low level during the convalescent phase of infection.

### Helper ILCs From febrile DHF Produce More cytokines Than Those of DF

To assess the functions of hILCs, we performed intracellular cytokine staining of hILCs from febrile DF and DHF patients, after *ex-vivo* stimulation. The expression of IFN-γ, IL-4 and IL-13, and IL-17A (representative functional cytokines of ILC1, ILC2, and ILC3, respectively) were evaluated on total hILCs (**Figures 3A, B**). In addition, IL-10 was also evaluated. The percentage of hILCs expressing IFN-γ was higher in DHF (median 23.85, IQR 15.05 - 28.43) than DF (median 14.42, IQR 12.94 - 19.92) (p = 0.042) (**Figure 3C**). Likewise, the percentage of hILCs producing IL-4 was also higher in DHF (median 7.86, IQR 6.98 - 11.94) compared to DF (median 5.23, IQR 4.08 - 6.19) (p = 0.003). A very small percentage of hILCs produce IL-17A (median 2.11 in DHF and median 1.21 in DF) (p = 0.036) (**Figure 3C**). No statistical differences in IL-13 and IL-10 production were observed among hILCs from DHF and DF (**Figure 3C**). This result suggests that hILCs from febrile DHF are functionally active and capable of producing their cytokines, more than in DF.

### Global Gene Expression Profiles of hILCs From DENV-Infected Patients

To explore the molecular functions of hILCs in febrile phase of DENV infection, we next examined the global gene expression profile using RNA-seq of FACS-sorted hILCs from samples of 3 DF patients, 3 DHF patients in febrile phase and matched convalescent phase of the same patients.

As expected, hILC signature genes, including *KLRB1* and *IL7R*, were detected in hILCs from all samples (**Figure 4A**). Meanwhile, the signature genes of hILC subsets (*GATA3*, *KIT*, *AHR*, *PTGDR2*, *TBX21*) were expressed at varying levels between samples (**Figure 4A**), likely due to the differences in the hILC subset composition among samples (**Figures 1C, D**). The gene markers for T cell, B cell, NK cell, and monocyte were rarely expressed in the hILCs. Together, these data further verified the identity of hILCs. Importantly, CD69 gene expression was upregulated in hILCs from febrile DF and febrile DHF, when compared to convalescence (**Figure 4B** lower panel), similarly to CD69 surface protein upregulation measured by flow cytometry (**Figure 4B** upper panel). Gene expression data from RNA-seq and protein expression data showed similar trends when compared between the two severities (DF vs DHF) and timepoints (febrile vs convalescence) (**Figure 4B**). Correlation analyses of global gene expression using unsupervised hierarchical clustering between samples showed that the febrile phase samples were clustered together (**Figure 4C**). However, the overall expression profiles cannot clearly distinguish the different disease severities. Principal component analysis (PCA) similarly showed hILCs from febrile phase samples clustered together and away from convalescent samples on PC2 (**Figure 4D**).

### Differential Gene Expression Analysis of hILCs From DENV-Infected Patients Across Severities and Timepoints

To further explore the functions of hILCs in febrile phase of DENV infection, we next performed pairwise differential gene expression analysis, comparing the differences between febrile and convalescent samples of the same patients in the DF and DHF groups (**Figure 5A** and **Supplementary Figures 1A, C**). Overall, a total of 261 and 228 genes were upregulated in the febrile phase of DF and DHF, respectively. Surprisingly, only 16 genes were upregulated in both DF and DHF, suggesting diverged functional responses of hILCs in different severity outcomes. Genes that were upregulated in the febrile phase of both DF and DHF, and those uniquely upregulated in DF or DHF are listed in **Supplementary Tables 4, 5**. We next performed functional gene set analysis using an over-representative test on the differentially expressed genes in the febrile DF (**Figure 5B**) and DHF (**Figure 5C**). In DF, the modules that were upregulated include pyruvate metabolic process, activation of JAK activity, positive regulation of TNF superfamily cytokine, and positive regulation of cytokine production (**Figure 5D**). In contrast, pathways that were upregulated in DHF include small molecule metabolic process, organophosphate and nucleotide/nucleoside biosynthetic process as well as nuclear mRNA surveillance of mRNA 3’-end process (**Figure 5E**).

To explore the diverse roles of hILCs during the febrile phase in DF and DHF with higher stringency, we applied shrinkage estimator ‘apeglm’ to analyse differential gene expression (85). After re-estimation of log2 fold change, only *EGR1* is upregulated in both febrile DF and DHF. The top immune-related genes uniquely upregulated in febrile DF (as compared to convalescence) were *IFIT1*, *IFI44L*, *HAVCR2* (Tim3) while those uniquely upregulated in febrile DHF were *GZMB*, *SLAMF7*, *RORC*, *TRIM21*, and *IL15RA*. (**Figure 5F**, **Supplementary Figure 1B, D** and **2** and **Supplementary Tables 6, 7**).

To corroborate the transcriptome analysis, we performed qPCR of *HAVCR2, GZMB, SLAMF7* and *TRIM21* from sorted hILCs as representatives of genes that were upregulated in DF or DHF. We observed a trend of higher *HAVCR2* expression in DF patients and higher *TRIM21* expressions in DHF patients (**Figure 5G**), as was observed in the transcriptome results. However, the expression of *GZMB* and *SLAMF7* were highly variable and no trend was observed between severities. The expression of *IFNG* as a representative cytokine gene of ILC1 was also show higher trend in DHF than DF (**Figure 5G**), consistently with the intracellular cytokine staining result. Taken together, our results suggest that in response to the DENV infection, hILCs were activated during the febrile phase in both DF and DHF, but demonstrated divergent transcriptomic responses, implying different functional roles in response to DENV infection.

## Discussion

To the best of our knowledge, this study showed the first evidence of hILC responses in human DENV infection, a pressing public health problem worldwide. By assessing blood samples from a well-characterized human DENV infection cohort, we found that hILCs were activated in the febrile phase of DENV infection and their cytokine production as well as transcriptional profiles in the febrile phase were distinct between DF and DHF patients.

Our results showed that the frequency of hILCs in the febrile phase of DENV infection remained unchanged when compared to matched convalescent samples, and to healthy control samples. This differs from acute HIV infection, where the number of circulating hILC were depleted irreversibly during acute infection likely from apoptosis (74). In animal models, hILC frequency was found to increase during some viral infections [IAV (56), rotavirus (67), late phase of MCMV (69), and rhinovirus (72)], but decrease in others [early phase of MCMV (69) and SIV (88)]. Although hILC frequency and subset composition remained unchanged in DENV infection, we found that hILCs were activated during the febrile phase. (**Figure 2**)

The activation of hILCs, as demonstrated by CD69 upregulation, was clearly observed during the febrile phase of DENV infection in both DF and DHF. ILC activation has also been observed in several other viral infections. For instance, lung ILC1 was previously shown to be activated in IAV infection along with the upregulation of IFN-γ (59). In addition, the upregulation of CD69 was found in circulating hILCs in early HIV infection (74). hILCs are generally known to be activated by cytokines depending on hILC subsets (32, 33). In IAV infection, lung ILC2 senses the alarmins from lung macrophages and subsequently secretes IL-13. In rotavirus infection, ILC3 responds to IL-1α secreted from intestinal epithelial cells (67). However, it is still unclear how hILCs are activated in the context of systemic viral infections, including DENV.

Our results showed that hILCs from the febrile phase of DHF produce more cytokines than those of DF. Cytokine storm is known to contribute to dengue severity (6, 9). While the higher serum level of IFN-γ and IL-4 have been previously observed in severe dengue patients (89), the cellular source of these cytokines especially IFN-γ are believed to be T cells (9, 90). We here showed that hILCs can secrete these cytokines during the febrile phase of infection, thus may contribute as the early source of these cytokines and likely play roles in shaping downstream adaptive T cell responses as previously shown in other models (91).

Our transcriptomic analysis suggests that the functions of hILCs likely differ between DF and DHF, as demonstrated by their distinct transcriptomic profiles. We first verified the sorted hILCs identity by observing high expression of hILCs combined gene sets, *PTPRC* (CD45), *IL7R* (CD127), and *KLRB1* (CD161), in all samples. We note some low level expression of conventional T/NK cell markers, *CD3E*, *GZMB*, and *SLAMF7*; these genes were also found to be lowly expressed in human hILCs in public transcriptomic datasets previously reported (**Supplementary Figure 3A**) (92). We next assessed transcriptomic profiles and observed clear distinction between hILCs from the febrile and convalescent phases, which were separated mainly by the expression of *CD69* and interferon-stimulated genes (*IFIT3*, *IFI44L*, *EGR1*), similarly to previous reports on whole PBMC transcriptome analysis in DENV-infected patients (93, 94). GO term analysis showed enrichment in different modules in the febrile DF and DHF. In DF, pyruvate metabolic process, activation of JAK activity, positive regulation of TNF superfamily cytokine, and positive regulation of cytokine production were upregulated, suggesting the activation of cytokine pathways, consistent with a previous report on whole blood transcriptome (95). On the other hand, upregulated genes in DHF were functionally enriched for organophosphate and nucleotide/nucleoside biosynthetic processes as well as nuclear mRNA surveillance of mRNA 3’-end processes. These suggest that hILCs were metabolically active and increased their transcription and translation. The activation of the transcription pathway was also found in ILC3 in HSV infection (66). Because the GO term analysis is relatively broad and not specific to hILC biology, we next examined DEG by focusing on immune-related genes that were upregulated in the febrile phase when compared to matched convalescent samples, especially those that are different between the febrile DF and DHF samples. Surprisingly, only *EGR1* was upregulated in both severities. *EGR1* is known to regulate IL-2 and TNFα production (96–98). In flavivirus infection, EGR1 was found to be commonly upregulated in T_EM_ and T_EMRA_ in both DENV and Zika virus infection (99). Furthermore, upon *in vitro* activation of T cells with DENV peptides, both activated CD4+ (100) and CD8+ T cells (101) upregulated *EGR1* expression. Thus, *EGR1* likely regulates proinflammatory cytokine production in hILCs during the febrile phase of DENV infection.

Our DEG analysis also suggests that hILC responses may be more tightly regulated in DF while relatively less so in DHF. In DF, febrile phase hILCs upregulated type I IFN response genes together with the negative regulators *IFI44L* and *HAVCR2* (Tim-3), suggestive of a “regulated response”. Interferon response gene, *IFIT1*, is known for its antiviral activity (102) while *IFI44L* is a feedback regulator of antiviral response (103). In human decidua, Tim-3 was found to be expressed in ILC3 and regulated IL-22, IL-8, and TNFα cytokine production important in maintaining feto-maternal tolerance (104). Tim-3, together with PD-1 and Tigit are considered check-point or inhibitory receptors on ILCs and NK (105). Quantification of Tim-3 expression by qPCR showed a similar trend of higher expression in DF but the difference did not reach statistical significance likely due to low number of samples. On the contrary, in the febrile phase of DHF samples, we observed upregulation of *TRIM21*, *GZMB*, *IL15RA*, and *SLAMF7* indicating pro-inflammatory response and metabolic activation. *TRIM21* was shown to respond to HCV and coxsackievirus B3 by interacting with MAVS (106). It sustains IRF3, thus positively regulates type I IFN antiviral response, and also activates proinflammatory cytokines TNFα, IL-6 (107, 108). Granzyme B is well known for its role in cytotoxic activity important in eradicating viral-infected cells (109). IL-15Rα, a receptor subunit for IL-15, is important for ILC development and activation especially in ILC1 (110). While *SLAMF7* function has not been characterized in hILCs, *SLAMF7* was found to express in ILC1 when we curated publicly available human circulating hILC RNA-seq data (92) (**Supplementary Figure 3A**). Interestingly, when we mapped the upregulated genes during the febrile phase of both DF and DHF, all were found to be relatively enriched in healthy ILC1 subset (**Supplementary Figure 3B**). This is consistent with the existing concept that ILC1 is the main ILC subset responding to viral infection. Taken together, hILCs likely participate in response to DENV through both cytokine-mediated response and cytotoxicity. Our data also suggest that a relatively loosely regulated activation of hILCs might be associated with severe DHF, while activation with regulated response may be associated with mild DF.

There are potential limitations of this study and future study is needed. First, we could not sort the hILC subsets separately for transcriptome analysis due to a limited number of hILCs in the samples. Future study using single-cell RNA sequencing could help elucidate the role of heterogeneous subsets of hILCs with higher resolution. In addition, future studies with a larger number of samples would be required to confirm our findings. Secondly, we could not detect hILC signature cytokine (IFN-γ, IL-4, IL-13, IL-17A) gene expression in RNA-seq data. This might be due to the low number of cells (150 cells per sample) as we observed a better, but not clear, expression of cytokine genes when we curated healthy hILC data from Li and colleagues (92) (**Supplementary Figure 3A**) which sorted 1,000 cells for RNA-seq experiment. In support of this hypothesis, another study with low number of cells also could not detect most of these genes in HIV infected hILCs data (74) (**Supplementary Figure 4**). Other possibilities include low cDNA conversion rate on these genes as well as insufficient sequencing depth. Nevertheless, intracellular cytokine staining and qPCR data helped to confirm that these cytokines were produced by hILCs during DENV infection. Finally, the cross-talks between hILCs and other cells are important aspects that are beyond the scope of the current study and warrant further investigations.

In summary, we provide the first evidence of hILC activation in human DENV infection and their distinct cytokine production and transcriptional profiles in the febrile phase of DF and DHF. While hILCs likely participate in antiviral defense against DENV infection, an uncontrolled response may be pathogenic and affect disease severity. Further investigations into the differential responses of hILCs in DENV infection will help us to better understand the protective response in DF and the pathogenic response in severe DHF. These could form the foundation for future applications, such as targeting pathogenic hILCs early in innate immune response to prevent disease progression to severe form, or harnessing hILCs to achieve optimally regulated immune response in vaccination strategy (111).

## Consortium

**DENFREE Thailand: Anavaj Sakuntabhai** (Functional Genetics of Infectious Diseases Unit, Institut Pasteur, Paris, France, Centre National de la Recherche Scientifique (CNRS), UMR2000, Paris, France), **Pratap Singhasivanon** (Department of Tropical Hygiene, Faculty of Tropical Medicine, Mahidol University, Bangkok, Thailand), **Swangjit Suraamornkul** (Endocrinology Division, Department of Medicine, Faculty of Medicine Vajira Hospital, Navamindradhiraj University, Bangkok, Thailand), **Tawatchai Yingtaweesak** (Thasongyang Hospital, Tak, Thailand), **Khajohnpong Manopwisedjaroen** (Department of Microbiology, Faculty of Science, Mahidol University, Bangkok, Thailand).

## Data Availability Statement

RNA-seq data discussed in this publication have been deposited in NCBI's Gene Expression Omnibus and are accessible through GEO Series accession number GSE155672. All RNA-seq analyses and visualizations were performed in R version 3.6. Computing code is available at github repository: https://github.com/vclabsysbio/hILCs_DENV.

## Ethics Statement

The studies involving human participants were reviewed and approved by Institutional Review Board of Faculty of Medicine Vajira Hospital (No.015/12), Faculty of Tropical Medicine Mahidol University (TMEC 13-041), and Faculty of Medicine, Ramathibodi hospital (COA.MURA2019/603). Written informed consent to participate in this study was provided by the participants’ legal guardian/next of kin.

## Author Contributions

PM conceptualized and designed the experiments. DENFREE Thailand provided clinical samples. PM and FL supervised wet lab experiments. TP performed the experiments with WC and AO support. TP performed bioinformatic analyses. VC and PM supervised bioinformatic analyses. PM, OM, VC, and TP analyzed and interpreted data. PM, OM, and TP wrote the manuscript. All authors contributed to the article and approved the submitted version.

## Funding

This work was supported by European Union Seventh Framework Program (EU/FP7 under Grant Agreement #282378 (DENFREE) to PM and DENFREE Thailand. The PM lab was supported by Anandamahidol foundation, Thailand Research Fund Grant for New Scholar (TRG5880121) and Royal Society-Newton Mobility Grant (NI170094) through the Office of Higher Education (Thailand) and the Royal Society (UK). The VC lab was supported by the Newton Advanced Fellowship through Thailand Research Fund (DBG60800003) and Royal Society (NA160153), and the Thailand Research Fund Grant for New Scholar (MRG6080235). TP was supported by the Scholarship for Young Scientists (2017), Faculty of Science, Mahidol University. This research project was supported by Mahidol University (Basic Research Fund: fiscal year 2021). WC was supported by The Royal Golden Jubilee (RGJ) Ph.D. Program Scholarship (Grant No. PHD/0115/2558) from Thailand Science Research and Innovation (TSRI).

## Conflict of Interest

The authors declare that the research was conducted in the absence of any commercial or financial relationships that could be construed as a potential conflict of interest.
