# Supplementary material for: Innate Lymphoid Cells Activation and Transcriptomic Changes in Response to Human Dengue Infection
Source: Front Immunol. 2021 May 17;12:599805. doi: 10.3389/fimmu.2021.599805 (PMC8165392; doi:10.3389/fimmu.2021.599805)
Supplement: Supplementary file 1 [file DataSheet_1.pdf]

Supplementary Materials

List of Supplementary Data

Supplementary Figure 1. Volcano plots showing differentially expressed genes comparing between febrile and convalescent phases in DF and DHF.
Supplementary Figure 2. Heatmap showing relative expression (TPM) of DE genes after applying 'apeglm' in each sample arranged by severity (DF, DHF) and timepoints (febrile, convalescence).
Supplementary Figure 3. Gene expression in public data from Li and colleagues (GSE112591).
Supplementary Figure 4. Gene expression in public data from Kløverpris and colleagues (GSE77088).
Supplementary Table 1. Characteristics of the DF and DHF patients whose blood samples were used in this study
Supplementary Table 2. List of antibodies used in flow cytometry experiments
Supplementary Table 3. List of primers used in qPCR experiments
Supplementary Table 4*. Differentially expressed genes comparing febrile and convalescent phase of DF patients.
Supplementary Table 5*. Differentially expressed genes comparing febrile and convalescent phase of DHF patients.
Supplementary Table 6*. Differentially expressed genes comparing febrile and convalescent phase of DF patients after applying 'apeglm' to re-estimate log2 fold change.
Supplementary Table 7*. Differentially expressed genes comparing febrile and convalescent phase of DHF patients after applying 'apeglm' to re-estimate log2 fold change.

* Supplementary Table 4-7 were uploaded in separated files

Supplementary Figure 1

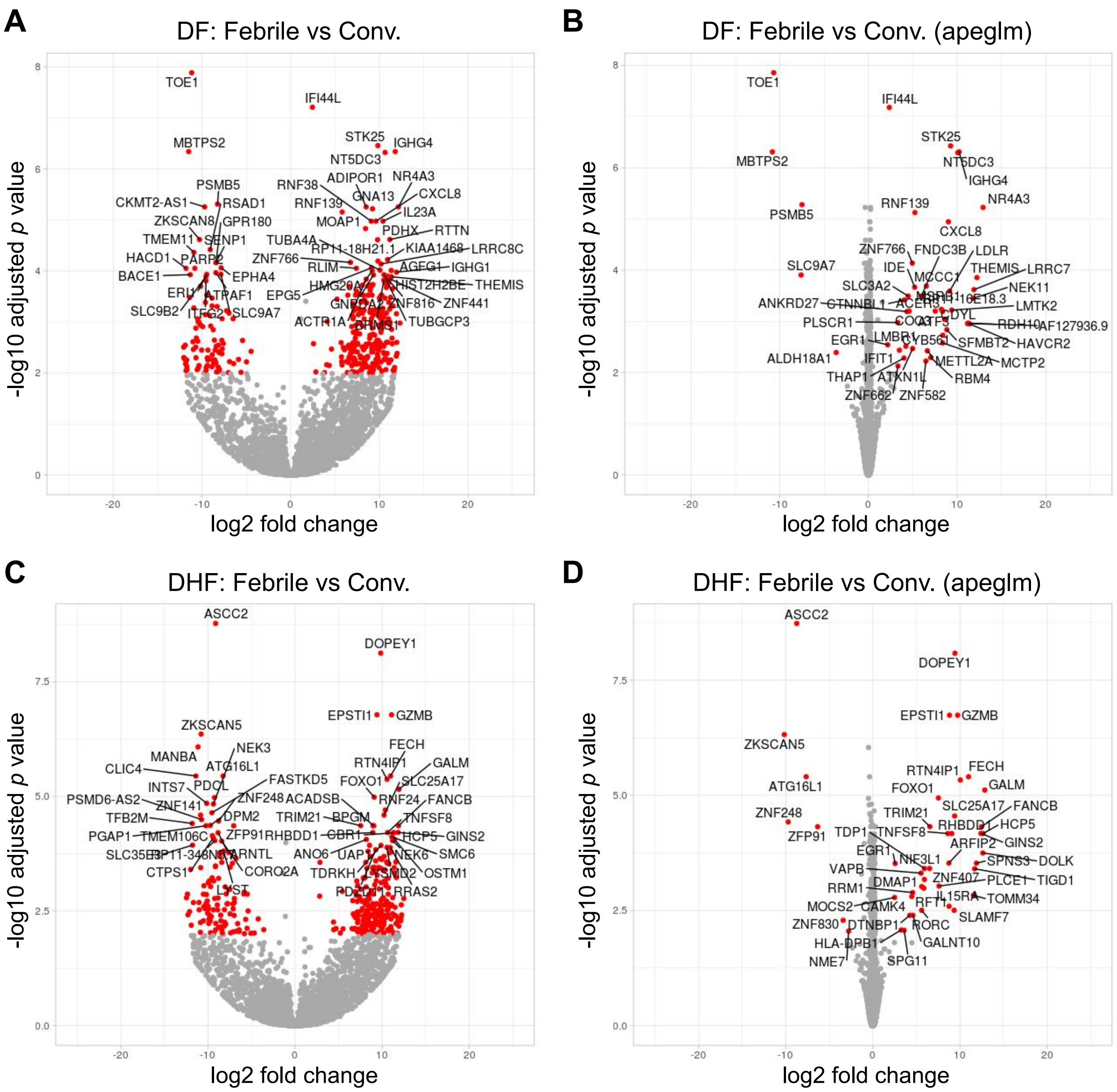

Supplementary Figure 1. Volcano plots showing differentially expressed genes comparing between febrile and convalescent phases in DF **(A, B)** and DHF **(C, D)**. DE analysis results from DESeq2 are plotted in (A,C) while volcano plots after applying 'apeglm' algorithm to re-estimate fold changes are shown in (B,D). Red dots highlighted statistically significant genes with adjusted *p* value < 0.01 and log2 fold change > 2. Only the top 50 significant genes are labelled in (A,C).

Supplementary Figure 2

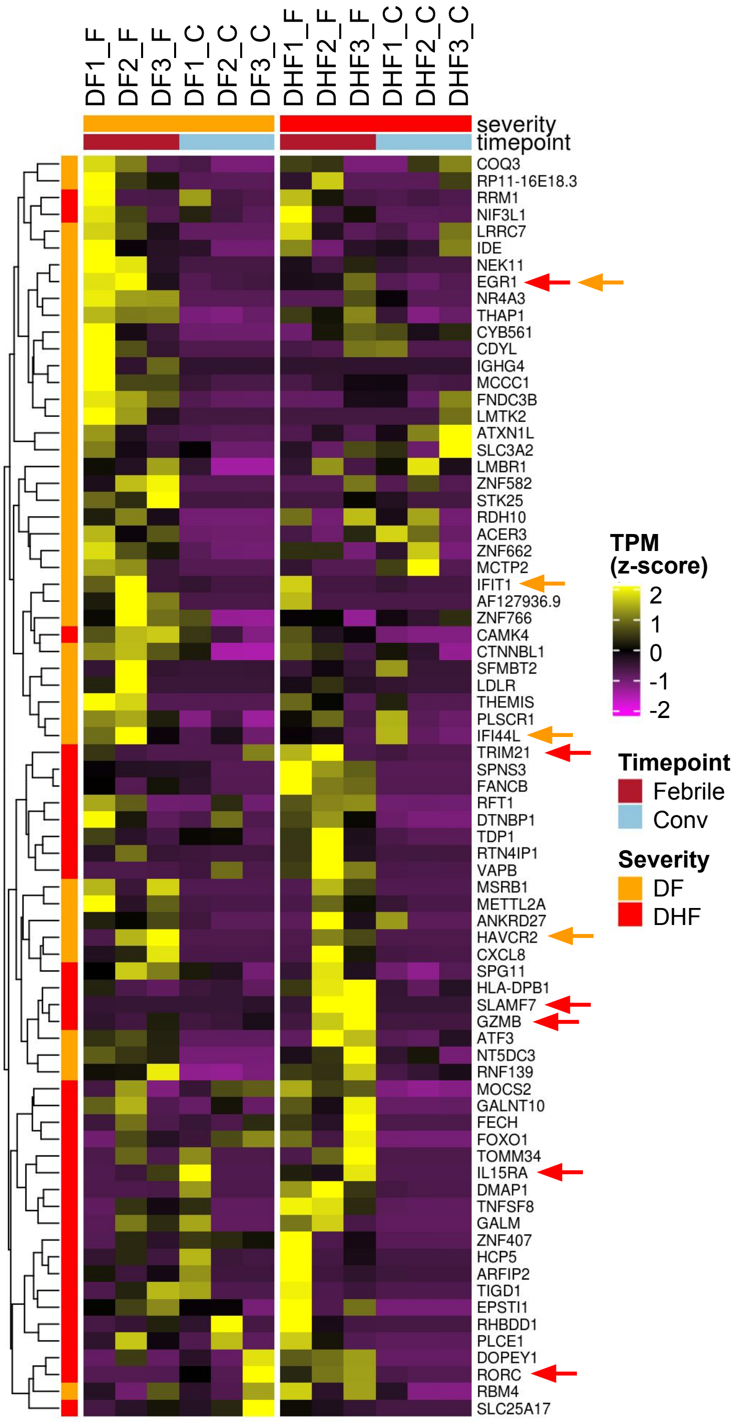

Supplementary Figure 2. Heatmap showing relative expression (TPM) of DE genes after applying ‘apeglm’ in each sample arranged by severity (DF, DHF) and timepoints (febrile, convalescence). Arrows indicate immune related genes significantly upregulated in febrile DF (orange) and DHF (red) as compared to paired convalescence samples.

Supplementary Figure 3

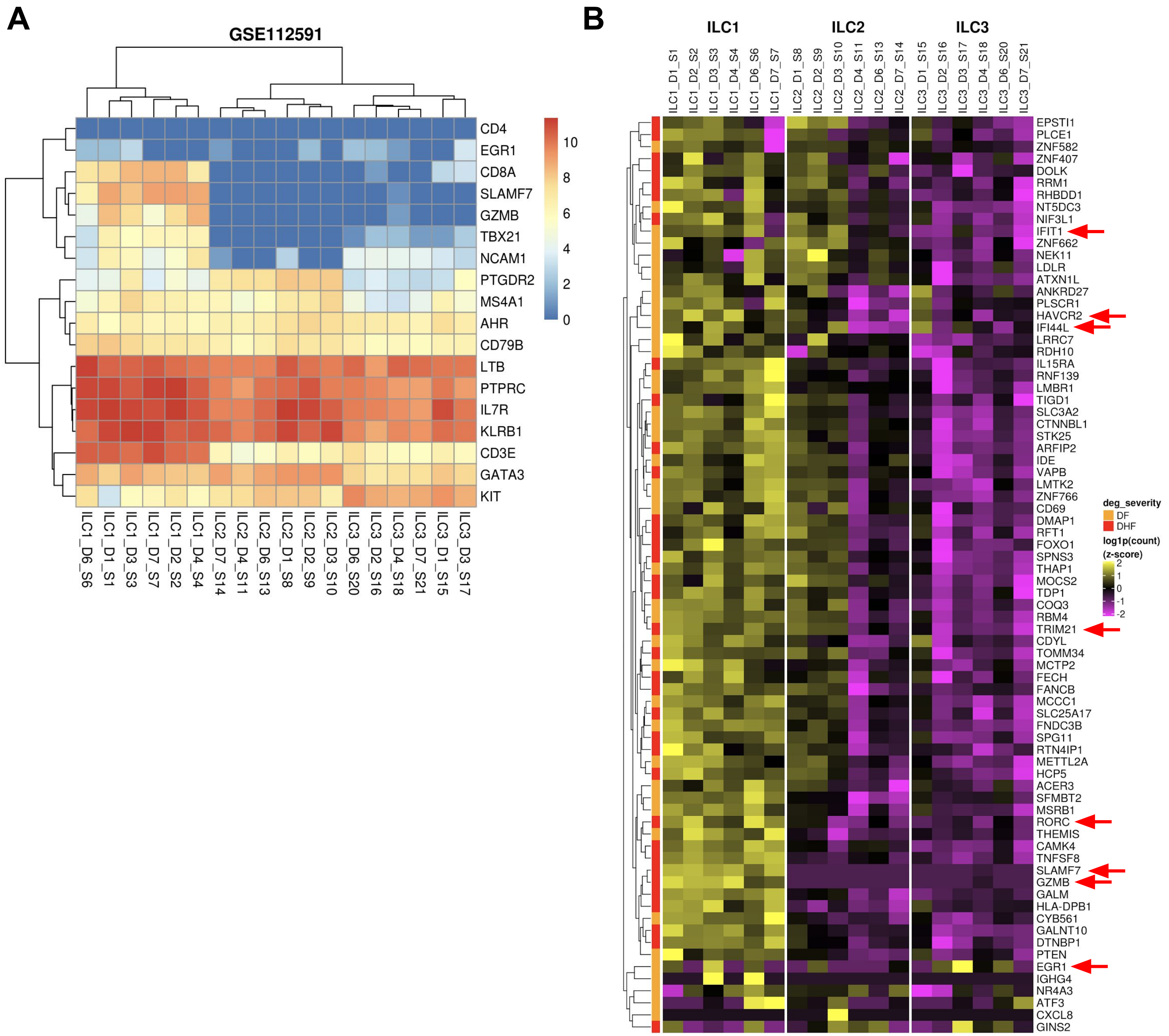

Supplementary Figure 3. Gene expression in public data from Li and colleagues (GSE112591). **(A)** Heatmap showing expression level of known hILC and their subsets signature genes. **(B)** Heatmap showing expression level of genes that are differentially expressed either in DF or DHF (as in Supplementary Figure 2) in GSE112591 healthy ILC subset dataset.

Supplementary Figure 4

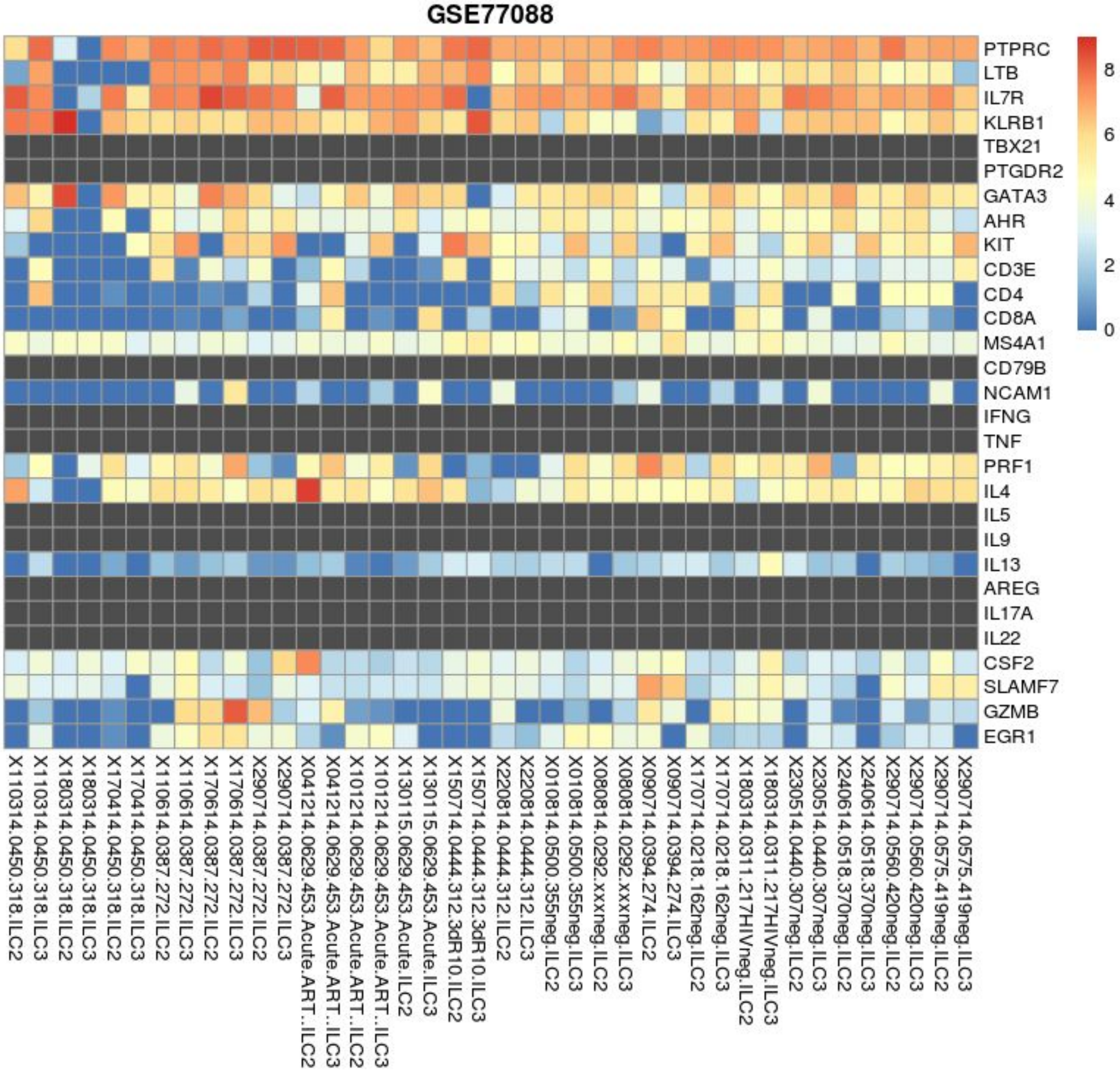

Supplementary Figure 4. Gene expression in public data from Kløverpris and colleagues (GSE77088). Heatmap showing expression level of known hILC and their subsets signature genes.

Supplementary Table 1

Surface flow cytometric experiment			
	**DF (n = 10)**	**DHF (n = 10)**	**P value (Mann–Whitney U test)**
Age, median in years (IQR)	31 (19.0-36.5)	22.5 (17.75-34.5)	0.75
Gender (% Male)	40.00%	60.00%	N/A
DENV viral load, median copies number per ml (IQR)	2.41E+05 (1.54E+04 - 5.86E+08)	7.54E+06 (8.61E+05 - 1.74E+07)	0.45
**Intracellular cytokine staining analysis experiment**			
	**DF (n = 10)**	**DHF (n = 14)**	**P value (Mann–Whitney U test)**
Age, median in years (IQR)	22 (19.0-32.0)	24 (19.0-27.0)	0.62
Gender (% Male)	40.00%	50.00%	N/A
DENV viral load, median copies number per ml (IQR)	1.25E+07 (1.22E+04 - 4.10E+07)	2.76E+05 (6.25E+03 - 5.03E+08)	0.73

Supplementary Table 1. Characteristics of the DF and DHF patients whose blood samples were used in this study

Supplementary Table 2

Reagents/Target	Florochrome	Vendor	Clones	Experiment	RRID
Human TruStain FcX™	-	Biolegend	-	Phenotyping/Sorting/ICS	BioLegend Cat# 422302
LIVE/DEAD™ Fixable Violet Dead Cell Stain Kit, for 405 nm excitatio	-	Invitrogen	-	Phenotyping	Invitrogen Cat# L34963
LIVE/DEAD™ Fixable Red Dead Cell Stain Kit, for 488 nm excitation	-	Invitrogen	-	Sorting	Invitrogen Cat# L34971
Zombie Violet™ Fixable Viability Kit	-	BioLegend	-	ICS	BioLegend Cat# 423114
Lineage Cocktail (CD3, CD14, CD16, CD19, CD20, CD56)	FITC	BioLegend	UCHT1, HCD14, 3G8, HIB19, 2H7, HCD56	Phenotyping/Sorting/ICS	BioLegend Cat# 348801
CD127 (IL-7Rα)	APC/Cy7	BioLegend	A019D5	Phenotyping	BioLegend Cat# 351348
CD127 (IL-7Rα)	APC/Fire750	BioLegend	A019D5	Sorting/ICS	BioLegend Cat# 351350
CD117	PE	BD Bioscience	YB5.B8	Phenotyping	BD Biosciences Cat# 561682
CD294	Alexa Fluor 647	BD Bioscience	BM16	Phenotyping	BD Biosciences Cat# 558042
CD69	Alexa Fluor 700	BD Bioscience	FN50	Phenotyping	BD Biosciences Cat# 560739
CD45	V500	BD Bioscience	HI30	Phenotyping	BD Biosciences Cat# 560777
IFN-γ	PE	BD Bioscience	4S.B3	ICS	BD Biosciences Cat# 559326
IL-4	Brilliant Violet 510	BioLegend	MP4-25D2	ICS	BioLegend Cat# 500836
IL-13	PerCP/Cy5.5	BioLegend	JES10-5A2	ICS	BioLegend Cat# 501911
IL-17A	Alexa Fluor 700	BioLegend	BL168	ICS	BioLegend Cat# 512318
IL-10	Alexa Fluor 647	BioLegend	JES3-9D7	ICS	BioLegend Cat# 501412

Supplementary Table 2. List of antibodies used in flow cytometry experiments (ICS = intracellular cytokine staining).

Supplementary Table 3

Gene	Forward Primer	Reverse Primer
GZMB	CCCTGGGAAAACACTCACACA	GCACAACTCAATGGTACTGTCG
HAVCR2	AGACAGTGGGATCTACTGCTG	CCTGGTGGTAAGCATCCTTGG
TRIM21	GTCCTGGAAAGGAGTGAGTCC	CTGAAAGTATCAGCCACGGATT
SLAMF7	TCAGGGATCTACTATGTGGGGA	GGTGCCATTCTTATTGCTCTGC
IFNG	TCGGTAACTGACTTGAATGTCCA	TCGCTTCCCTGTTTTAGCTGC
ACTB	CATGTACGTTGCTATCCAGGC	CTCCTTAATGTCACGCACGAT

Supplementary Table 3. List of primers used in quantitative PCR experiments
